# Synthesis and Cytotoxicity Studies of Wood-Based Cationic Cellulose Nanocrystals as Potential Immunomodulators

**DOI:** 10.3390/nano10081603

**Published:** 2020-08-15

**Authors:** Yusha Imtiaz, Beza Tuga, Christopher W. Smith, Alexander Rabideau, Long Nguyen, Yali Liu, Sabahudin Hrapovic, Karina Ckless, Rajesh Sunasee

**Affiliations:** 1 Department of Chemistry, State University of New York at Plattsburgh, Plattsburgh, New York, NY 12901, USA; yimti001@plattsburgh.edu (Y.I.); btuga001@plattsburgh.edu (B.T.); c.w.smith022@gmail.com (C.W.S.); arabi005@plattsburgh.edu (A.R.); lnguy007@plattsburgh.edu (L.N.); 2 Aquatic and Crop Resource Development Research Centre, National Research Council Canada, Montreal, QC H4P 2R2, Canada; yali.liu@cnrc-nrc.gc.ca (Y.L.); sabahudin.hrapovic@cnrc-nrc.gc.ca (S.H.)

**Keywords:** cellulose nanocrystals, immunomodulator, synthesis, polymerization, characterization, cytotoxicity

## Abstract

Polysaccharides have been shown to have immunomodulatory properties. Modulation of the immune system plays a crucial role in physiological processes as well as in the treatment and/or prevention of autoimmune and infectious diseases. Cellulose nanocrystals (CNCs) are derived from cellulose, the most abundant polysaccharide on the earth. CNCs are an emerging class of crystalline nanomaterials with exceptional physico-chemical properties for high-end applications and commercialization prospects. The aim of this study was to design, synthesize, and evaluate the cytotoxicity of a series of biocompatible, wood-based, cationic CNCs as potential immunomodulators. The anionic CNCs were rendered cationic by grafting with cationic polymers having pendant ^+^NMe_3_ and ^+^NH_3_ moieties. The success of the synthesis of the cationic CNCs was evidenced by Fourier transform infrared spectroscopy, dynamic light scattering, zeta potential, and elemental analysis. No modification in the nanocrystals rod-like shape was observed in transmission electron microscopy and atomic force microscopy analyses. Cytotoxicity studies using three different cell-based assays (MTT, Neutral Red, and LIVE/DEAD^®^) and three relevant mouse and human immune cells indicated very low cytotoxicity of the cationic CNCs in all tested experimental conditions. Overall, our results showed that cationic CNCs are suitable to be further investigated as immunomodulators and potential vaccine nanoadjuvants.

## 1. Introduction

Polysaccharides, such as 2,3-O-acetylated-1,4-β-D-glucomannan, have been shown to have immunomodulatory properties, including the stimulation of cytokines, interleukin 1-beta (IL–1β), and tumor necrosis alpha (TNF-α) in human cell lines [1,2]. The immune system is a complex network of different cell types and mediators responsible for the defense of an organism against pathogens as well as the maintenance of tolerance to innocuous antigens [3]. Immunomodulation plays a crucial role in physiological processes as well as in the treatment and/or prevention of several diseases, including infectious diseases. Therefore, studies focusing on polysaccharide-based nanomaterials with immunomodulatory activities can have an important impact on development of novel vaccine adjuvants. Adjuvants, in the context of vaccines, are defined as components capable of enhancing and/or shaping antigen-specific immune responses [4]. Advances in particle engineering allow the design of new nanomaterials with desired physico-chemical properties, such as composition, size, shape, surface characteristics, and degradability [5], which ultimately will impact their effect as immunomodulators. It is well known that cationic substances, especially particulate materials, can act as immunomodulators [6,7]. In fact, the immunostimulatory activity of cationic cellulose nanocrystals (CNCs) was first described in our previous work in which we found that they induced the secretion of the inflammatory cytokine, IL-1β, in mouse and human macrophage cells [8,9]. CNCs are elongated crystalline rod-like (or needle-like) nanoparticles derived from the most abundant natural biopolymer on earth, cellulose [10,11,12]. CNCs are green and biocompatible materials with unique physico-chemical properties, including high aspect ratio, low density, large specific surface area, tunable surface chemistry (presence of abundant hydroxyl groups), non-toxic, colloidal stability, and optical and mechanical properties. These favorable features make CNCs as attractive nanoscale materials for applications in various fields such as biomedical, pharmaceutical, nanocomposites, electronics, among many others [13,14,15]. CNCs can be manufactured on a large scale and a recent study showed that the current standing of industrially produced CNCs was positive, indicating that the evolution of commercial-scale applications would not be hindered by CNCs production [16]. The emerging interest in CNCs for biomedical purposes led us to synthesize a novel functionalized series of wood-based CNCs possessing surface positive charges with potential immunomodulatory activities that could hopefully be further developed in newly engineered vaccine nanoadjuvants. For applications that required a positively charged CNCs surface, synthetic routes for cationization of CNCs were developed such as nucleophilic ring-opening of epoxide, esterification, copper(I)-catalyzed 1,3-cycloaddition and surface initiated-atom transfer radical polymerization [17,18,19,20,21,22]. In this work, we reported the design, synthesis, characterization and cytotoxicity of wood-based cationic CNCs as potential immunomodulators. A series of colloidally stable cationic CNCs was prepared via surface-initiated single electron transfer living radical polymerization (SI-SET-LRP) technique with different sizes and surface charges. The choice of [2-(methacryloyloxy)ethyl] trimethylammonium chloride (METAC) and aminoethyl methacrylate hydrochloride (AEM) monomers with pendant cationic groups (+NMe_3_ and + NH_3_ respectively) was guided in part due to a recent study highlighting the use of NH_2_-functionalized aluminum oxyhydroxide nanorods for an enhanced immune adjuvant activity [7]. In addition to an effective synthetic route and sound physico-chemical characterization, another important aspect for the potential application of these cationic CNCs as vaccine nanoadjuvant is safety of these nanomaterials. In general, vaccine adjuvant should not induce an overwhelming immune response, otherwise it would have a toxic effect. Therefore, the cytotoxicity of these cationic CNCs was evaluated using three different cell-based assays (MTT, Neutral Red, and LIVE/DEAD^®^) and relevant immune cells, including two mouse cell lines (J774A.1 and BV2) as well as human peripheral blood mononuclear cells (PBMCs). Overall, we successfully synthesized and characterized a series of cationic CNCs that showed very low cytotoxicity in all tested experimental conditions indicating that these cationic cellulose-based nanomaterials are suitable to be further investigated as immunomodulators and potential vaccine nanoadjuvants.

## 2. Materials and Methods

### 2.1. Materials and Reagents

Spray-dried sulfated CNCs (prepared by sulfuric acid mediated hydrolysis of hardwood pulp) was kindly supplied by InnoTech Alberta Inc (Edmonton, AB, Canada). The spray-dried CNC powder was stored at 4 °C and used as obtained. Triethylamine (TEA), 4-dimethyl-aminopyridine (DMAP), 2-bromoisobutyryl bromide, *N*,*N*,*N*′,*N*″,*N*″-pentamethyldiethylenetriamine (PMDETA), copper (I) bromide, [2-(methacryloyloxy)ethyl] trimethylammonium chloride (METAC) (75 wt% in H_2_O), 2-aminoethyl methacrylate hydrochloride (AEM), 2,2′-bipyridyl (bpy), methanol, ethanol, tetrahydrofuran (THF), acetone, and potassium bromide (KBr) were purchased from Sigma-Aldrich (St Louis, MO, USA).

### 2.2. Synthesis of Wood-Based Cationic CNCs

The initiator modified CNC materials (CNC-BriB) were first synthesized according to our previously reported procedure with slight modifications [23]. Pristine spray-dried CNCs were reacted with 2-bromoisobutyryl bromide (BriB bromide) at two different ratios with respect to anhydroglucose units (AGU) in CNCs ([Br]/[AGU]), 5:3 for CNC-BriB-1 and 5:12 for CNC-BriB-2 (for a detailed experimental procedure of CNC-BriB, refer to SI & Supplementary Figure S1). CNC-BriB was then grafted with either poly (METAC) or poly (AEM) via SI-SET-LRP at different ratios of monomer to AGU such as [METAC/AGU] = 50:3 or 60:3 and [AEM/AGU] = 50:3 (Table 1). A general procedure for the preparation of cationic CNCs is as follows: CNC-BriB (350 mg) was dispersed in a MeOH:H_2_O solvent mixture (100 mL, 1:1 v/v) in a 250 mL Schlenk flask. The reaction mixture was degassed under N_2_ gas for 45 min prior to the addition of the monomer METAC or AEM (50 and/or 60 mmol) and copper (I) bromide (0.5 mmol). The suspension was degassed again before addition of bpy or PMDETA (1 mmol) and the reaction mixture was vigorously stirred at room temperature for 24 h. The resulting crude cationic CNC was then centrifuged (3 × 12,000 rpm at 10 °C for 30 min) with a 1:3 ratio of H_2_O:MeOH. The residual solid was resuspended in water and extensively dialyzed (MWCO 3500 Da) against deionized water for 1 week with daily constant change of water. The suspension was then freeze-dried to afford purified CNCs as white flaky material. The syntheses of other cationic CNCs are described in detail in the supporting information and Supplementary Table S1.

### 2.3. Characterization of Pristine and Modified CNCs

#### 2.3.1. Fourier Transform Infrared Spectroscopy (FTIR)

FTIR spectra of pristine spray dried sulfated CNCs and lyophilized esterified and cationic CNCs were recorded on a PerkinElmer FTIR spectrophotometer (Spectrum Two) (Norwalk, CT, USA) at room temperature. KBr pellets were prepared by grinding in a mortar and compressing about 2% of the CNC samples in KBr (previously well-dried in an oven). Background measurement using a neat KBr pellet was first obtained to correct for light scattering losses in the pellet and any water absorbed by KBr. Spectra in the range of 4000–400 cm^−1^ were obtained with a resolution of 4 cm^−1^ by cumulating 32 scans.

#### 2.3.2. Zeta Potential and Dynamic Light Scattering

Zeta (ζ-) potential and dynamic light scattering (DLS) measurements were conducted using a Malvern Zetasizer Nano ZS instrument (model: ZEN3600; Malvern Instruments Inc., Westborough, MA, USA). This instrument is equipped with a 4.0 mW helium-neon laser (λ=633 nm) and an avalanche photodiode detector and works at a 173° scattering angle. A 0.25 wt% CNC dispersion in Milli-Q water was used for zeta potential while for DLS, the hydrodynamic apparent particle size was measured for a 0.05 wt% CNC dispersion in Milli-Q water. Prior measurements, the suspensions were sonicated and filtered through a 0.45 µm filter membrane and analyses were performed immediately at 25 °C. For DLS results, triplicate samples were measured 12 times each and the average particle size distribution was obtained. The standard deviation was reported as the error for the measurements. Results for zeta potential measurements were recorded in triplicate and the averages were reported.

#### 2.3.3. Elemental Analysis

Carbon, hydrogen, nitrogen, and sulfur content by mass for freeze-dried pristine and cationic CNC samples were determined using Thermo Scientific Flash 2000 Organic Elemental Analyzer. Analysis (Edmonton, AB, Canada) was performed in duplicate and the averages were reported.

#### 2.3.4. Transmission Electron Microscopy

Transmission electron microscopy (TEM) images of pristine and cationic CNC samples were obtained using Hitachi H-7500 operating at 80 kV in HR mode. All samples were prepared at 0.1 mg/mL in double distilled water and bath sonicated for 15 min at room temperature prior to the sample immobilization on TEM grids. 6 µL of sample were deposited on the TEM grid (Cu-300CN, Pacific Grid-Tech, San Francisco, CA, USA) with excess solution removed by using the edge of a wet filter paper. Samples were stained in 1 wt% phosphotungstic acid (PTA) in water (pH 7) for 60 s and air-dried in the dark prior to the TEM analysis. The samples were observed in the magnification range of 40,000–600,000 Å in order to provide enough information about sample purity and morphology.

#### 2.3.5. Atomic Force Microscopy

Atomic force microscopy (AFM) micrographs of pristine and cationic CNCs were obtained using a Nanoscope IV (Digital Instruments Veeco, Santa Barbara, CA, USA) with a silicon tip operated in tapping mode. Particle analysis of the micrographs was performed using the software Scanning Probe Image Processor^TM^ (5.0.8.0; Image Metrology, DK-2800 Lyngby, Denmark)

### 2.4. Cytotoxicity Studies

#### 2.4.1. Preparation of the Colloidal Suspension of CNCs for Cell-Based Assays

For cytotoxicity assays, the CNC suspensions were prepared at 1 mg/mL in ultrapure water. The CNC suspensions were vortexed for 15 s, sonicated for 2 min at 70 output, followed by filtration with 0.45 µm polytetrafluoroethylene (PTFE) filter and autoclaved at 121 °C, 15 psi for 30 min. The suspensions were aliquoted and kept at −20 °C for future use.

#### 2.4.2. Cell Culture and Experimental Conditions

Mouse macrophage-like cell line (J774A.1, Sigma) and BV2 cell line (murine microglia, kindly provided by Dr. Amy Ryan, SUNY Plattsburgh) were seeded at 3–5 × 10^5^ cells/mL in 96 well plate (3–5 × 10^4^ cells/well) using RPMI 1640 and DMEM medium (GIBCO), respectively. Both media were supplemented with 10% fetal bovine serum (FBS), penicillin, streptomycin, and l-glutamine. Both cell lines were cultured at 37 °C in a 5% CO_2_-supplemented atmosphere for at least overnight before the treatment with 10, 25, 50, and 100 µg/mL of CNCs. The peripheral blood mononuclear cells (PBMCs) were extracted from Leukotrap blood filters from healthy blood donors. The Leukotrap filters were obtained from UVM Health Network-CVPH North Country Regional Blood Center, Plattsburgh, NY. To retrieve blood cells, the filter was reverse flushed with 3 × 50 mL of calcium and magnesium-free PBS, followed by PBMCs isolation using a separation medium (LSM). Briefly, 4 mL of LSM was added to a 15 mL conical centrifuge tube and 6 mL of 1:1 diluted blood in calcium and magnesium-free PBS was carefully layered on LSM, creating a sharp blood-LSM interface. The conical tubes were centrifuged at 400× *g* at room temperature for 25 min. The top layer containing plasma was discarded and the mononuclear cell layer was carefully transferred to a clean tube and an equal volume of calcium and magnesium-free PBS was added. The cell suspension was centrifuged at 250× *g* to pellet cells and the cells were washed once in calcium and magnesium-free PBS and finally resuspended in RPMI medium for seeding. The PBMCs were seeded in 48 well plate at 1×10^6^ cells/mL and cultured at 37 °C in a 5% CO_2_-supplemented atmosphere for at least overnight before the treatment with 10, 25, 50, and 100 µg/mL of CNC derivatives.

#### 2.4.3. Cell Viability Assays

After 24 h of treatment, the medium from J774A.1 and BV2 treated and non-treated (control) cells was removed, and 100 µL of fresh culture medium containing 500 µg/mL of 3-(4,5-dimethylthiazol-2-yl)-2,5-diphenyltetrazolium bromide (MTT) or 50 µg/mL of Neutral Red (NR) was added to each well to access cell viability. The MTT assay involves the conversion of the water soluble MTT (yellow) by mitochondrial dehydrogenases in viable cells to a water-insoluble formazan (purple/blue) [24] while the NR assay is based on the ability of viable cells to incorporate and bind the dye neutral red in the lysosomes [25]. In both assays, the intensity of the color is directly proportional to cell viability. The cells with MTT or NR loading medium were incubated for at 37 °C in a 5% CO_2_-supplemented atmosphere. After 30 min, the respective loading medium was removed, and the attached cells were gently washed once with PBS. To solubilize the formazan crystals, 100 μL/well of dimethyl sulfoxide (DMSO) was added and the absorbance was measured at 570 nm using a microplate reader (Synergy H1 Hybrid Multi-Mode, BioTek/Agilent, Winoosky, VT, USA). To extract NR dye from lysosomes, 100 μL of acidified ethanol (1% glacial acetic acid, 50% aqueous ethanol) was added and the plate was placed on the plate shaker for ~10–15 min with protection from light. The absorbance at 540 nm and at 690 nm was measured in the microplate reader within 60 min of adding NR Desorb solution. For calculations, 690 nm was subtracted from 540 nm. The non-treated cells (control) were considered 100% of viable cells in both MTT and NR assays. For statistical significance, both cytotoxicity assays were repeated at least 3 times in triplicates.

The LIVE/DEAD^®^ viability assay is a quick and easy two-color assay to determine the viability of cells in a population. Typical results will distinguish live cells which are stained with green-fluorescent calcein-AM to indicate intracellular esterase activity, from dead cells that are stained with red-fluorescent ethidium homodimer-1 to indicate loss of plasma membrane integrity. The assay was performed according to the manufacturer’s instructions. Briefly, after 24 h treatment with respective CNCs, the cell culture medium was gently removed and 250 µL PBS, 2 µL of Calcein (40 µM) and 2 µL of ethidium homodimer-1 2 mM were added, mixed, and the cell suspension was incubated in the dark RT for 15 min. The loading solution was discarded and 250 µL of cold PBS + EDTA was added to each well and the plate was placed on ice for 30 min to facilitate the release of PBMCs from the plate. The bottom of the wells was scraped with a pipette tip and the cells were removed by gentle pipetting up/down, 100 µL of the cell suspension was utilized to assess live and dead cells by flow cytometry (BD Accuri™ C6 cytometer, BD, Franklin Lake, NJ, USA) using FL-1 filter Calcein (green, live) and FL-2 filter Ethidium homodimer (red, dead). The percentage of labeled cells was calculated by BD Accuri software using H_2_O_2_ treated cells as positive control for gating dead cells. The calculated data was expressed using the average of fold of change in the (%) of dead cells (ethidium bromide staining) corrected by (%) dead cells in the control (non-treated cells), from at least 4 experiments.

#### 2.4.4. Statistical Analysis

The data were statistically analyzed by using the two-way analysis of variance test followed by Dunnett’s multiple comparison test using GraphPad Prism version 8.2 software (San Diego, CA, USA). Statistical significance was defined as *p* < 0.01.

## 3. Results and Discussion

### 3.1. Synthesis and Characterization of Cationic CNCs

**Scheme 1:**
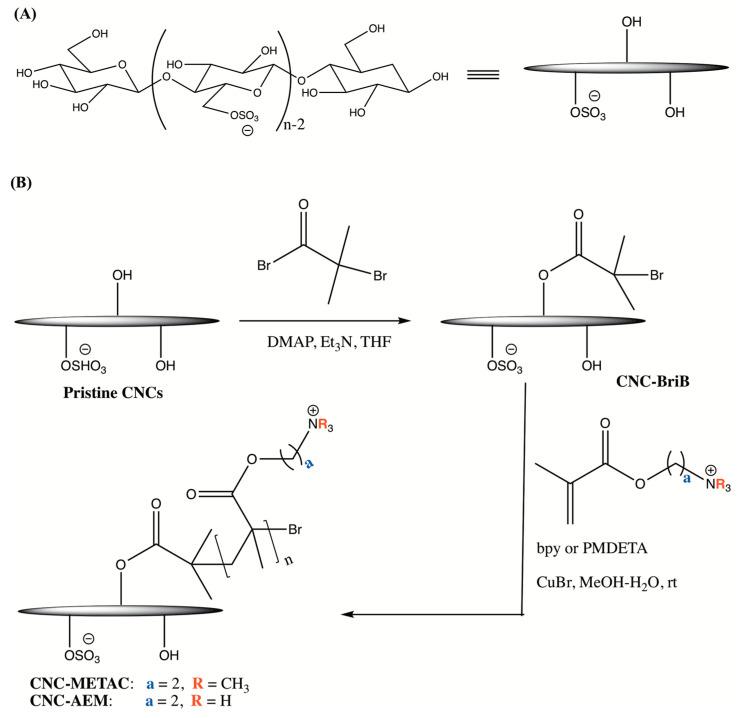
(**A**) chemical structure and schematic representation of pristine CNCs; (**B**) synthetic strategy for the fabrication of a series of cationic CNCs.

CNCs with cationic polymer brushes were synthesized by grafting poly (METAC) or poly(AEM) via SI-SET-LRP from the surface of pristine CNCs (Scheme 1). The first step required the covalent attachment of an initiator on the surface of CNCs using a well-known esterification method of the hydroxyl groups of CNCs with an acyl bromide [23,26,27]. CNC-initiator (CNC-BriB) were prepared at two different initiator loading capacity with respect to anhydroglucose units (AGU) in CNCs ([Br]/[AGU]), 5:3 for CNC-BriB-1 and 5:12 for CNC-BriB-2). The next key step involved a grafting from approach to introduce cationic poly(METAC) or poly(AEM) brushes by SI-SET-LRP on CNCs under mild reaction conditions (Scheme 1B). SET-LRP is a robust and versatile polymerization technique for materials synthesis [28] and had been previously used to graft thermoresponsive and methacrylamide-based polymers from the surface of CNCs [23,26,27,29]. A series of cationic CNCs was synthesized by varying the ratio between the immobilized initiator and the monomers (Table 1). After the polymerization, the resulting cationic CNCs were extensively purified by several centrifugations and dialysis over a week to ensure the removal of unreacted materials or any trace impurities.

**Figure 1:**
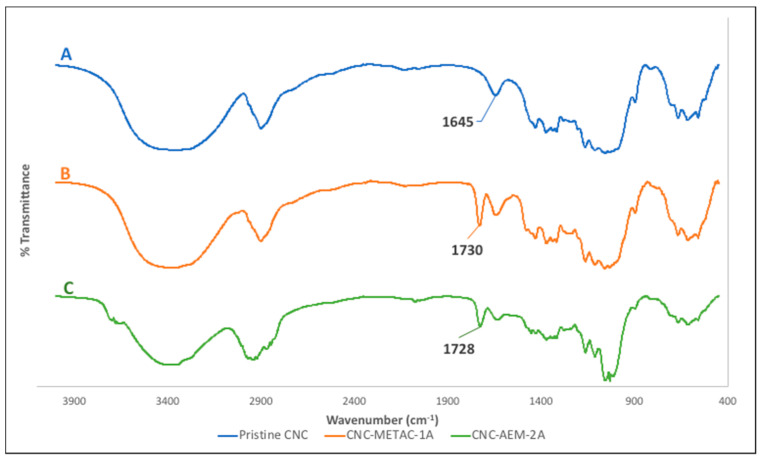
FTIR spectra of cationic CNCs: (A) pristine CNC, (B) CNC-METAC-1A, (C) CNC-AEM-2A.

The success of the grafting of cationic brushes on the surface of CNCs was confirmed by FTIR spectroscopy, DLS, zeta potential and elemental analysis. Pristine CNCs displayed typical IR peaks characteristic of cellulosic functional groups at 3000–3600 cm^−1^, 1645 cm^−1^ and 900–1150 cm^−1^ corresponding to the -OH, -OH bending of water and -C-O-C vibrations, respectively [30,31] (Figure 1, spectrum A; Supplementary Figure S2). The spectra of CNCs after the polymerization showed a notable change with the appearance of new IR peaks at 1730 cm^−1^ and 1728 cm^−1^ for CNC-METAC-1A (Figure 1, spectrum B) and CNC-AEM-2A (Figure 1, spectrum C), respectively. These peaks were attributed to the typical carbonyl stretching vibration (C = O) of ester moieties confirming the presence of poly (METAC) and poly (AEM) on the surface of CNCs [27].

**Figure 2:**
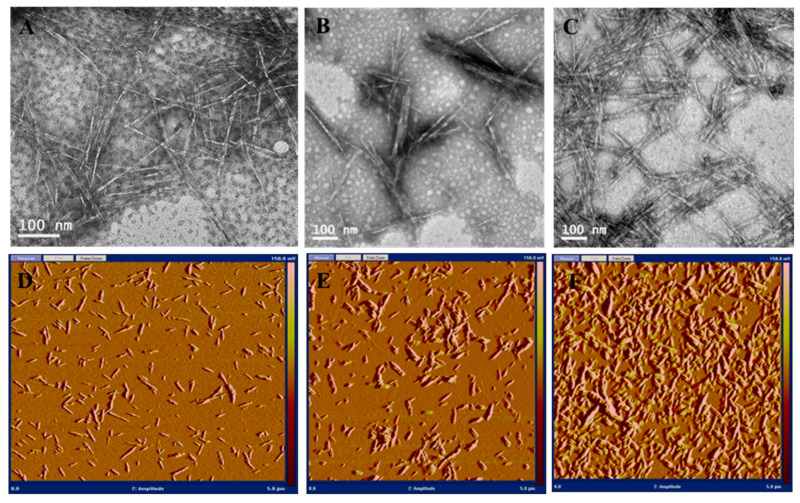
Morphological analyses of pristine and cationic CNCs by TEM and AFM: Representative TEM images of (**A**) pristine CNCs; (**B**) CNC-METAC-1A; (**C**) CNC-METAC-1B and AFM phase images of (**D**) pristine CNCs, (**E**) CNC- METAC-2A; (**F**) CNC-METAC-2B. TEM and AFM images of other cationic CNCs are depicted in Figures S4 and S5.

Dynamic light scattering (DLS) and zeta potential measurements were conducted to explore the surface properties and stability of CNC suspensions before and after polymer grafting. Although the DLS technique is used for spherical particles, the measurements have been commonly employed to compare the relative sizes of rod-like CNCs before and after a chemical modification as well as the state of dispersion of the particles [16,21]. However, particle size measurements by DLS are not absolute and should not be considered as the exact dimensions of pristine and modified CNCs. The apparent hydrodynamic diameter of CNC particles was obtained by DLS in water at a 0.05 wt% concentration. Pristine CNCs showed an apparent particle size of ~101 nm which is in close agreement with the data obtained from AFM analysis (Figure 2D). An increase in apparent particle size was observed for all cationic CNCs (Table 2, Supplementary Figure S3) which indicated the presence of the polymer brushes on the surface of CNCs and as expected, the cationic CNCs would be highly hydrated. Furthermore, DLS analysis indicated no apparent aggregation of CNC particles at low concentrations. The nature of the surface charges on pristine and cationic CNCs was examined by measuring the zeta potential of the particles in water at 0.25 wt% concentration. Pristine CNCs had a negative zeta potential (-34.8 mV) due to the presence of anionic sulfate half-ester groups. On the other hand, cationic CNCs displayed positive zeta potential values in the range of +31.8 to +45.0 mV (Table 2). This further confirmed a successful polymerization reaction and the grafted cationic polymer brushes shielded the anionic sulfate half-ester groups. An increase in zeta potential (becomes more positive) was observed with an increase in monomer concentration ([monomer/AGU]: 50:3 v/s 60:3, Table 1). For instance, CNC-METAC-1B which had a higher monomer concentration compared to CNC-METAC-1A showed a more positive zeta potential value (+44.9 mV v/s +31.8 mV). The same trend was observed for CNC-METAC-2B (+38.2 mV) compared to CNC-METAC-2A (+32.0 mV). Overall, the cationic nature and colloidal stability of CNC-METAC and CNC-AEM materials are crucial for consistent results in biological studies.

The atomic composition of CNC samples before and after polymer grafting was measured by elemental analysis (Table 3). As expected, a small percentage of sulfur was detected for pristine CNCs due to the presence of the sulfate half-ester groups. While pristine CNCs displayed the absence of nitrogen, all the prepared cationic CNCs indicated the appearance of nitrogen deriving from the cationic monomers (METAC or AEM). This further supported the success of the polymerization reaction. The cationic CNC with a fixed initiator content and increased monomer concentration (CNC-METAC-1B v/s CNC-METAC-1A, Table 1) had a higher nitrogen content (~7.68%).

The morphological features of the cationic CNCs were analyzed by both transmission electron microscopy (TEM) and atomic force microscopy (AFM) to verify whether the characteristic rod-like crystalline structure of pristine CNCs (Figure 2A,D) was maintained after the surface modification reactions. Figure 2B–F, Figures S4 and S5 indicated that the rod-like morphology of the cationic CNCs was retained and the samples did not degrade into more simple carbohydrates [32]. Accurate size measurement of the rod-like particles by TEM was difficult as the edges of the grafted CNCs were less defined as well as a tendency to aggregate during air-drying or staining [33]. AFM analysis of pristine CNCs depicted a good distribution of the nanorods possessing an average length of 104 ± 68 nm and diameter of 4.0 ± 0.5 nm with a resultant aspect ratio of 26 (Figure 2D). The size of the cationic CNCs was found to be in the range of 112–164 nm in average length and 4.9–5.3 nm in cross-section. Moreover, AFM phase images of the cationic CNCs (Figure 2D,E) showed a halo that was indicative of the presence of grafted polymer brushes [26]. Aggregation of nanoparticles was observed for CNC-METAC-2B (Figure 2F) as it was prepared with a high monomer concentration (compared to CNC-METAC-2A (Figure 2E)), and hence a high degree of polymerization was expected [34].

### 3.2. Cytotoxicity Studies of Cationic CNCs

**Figure 3:**
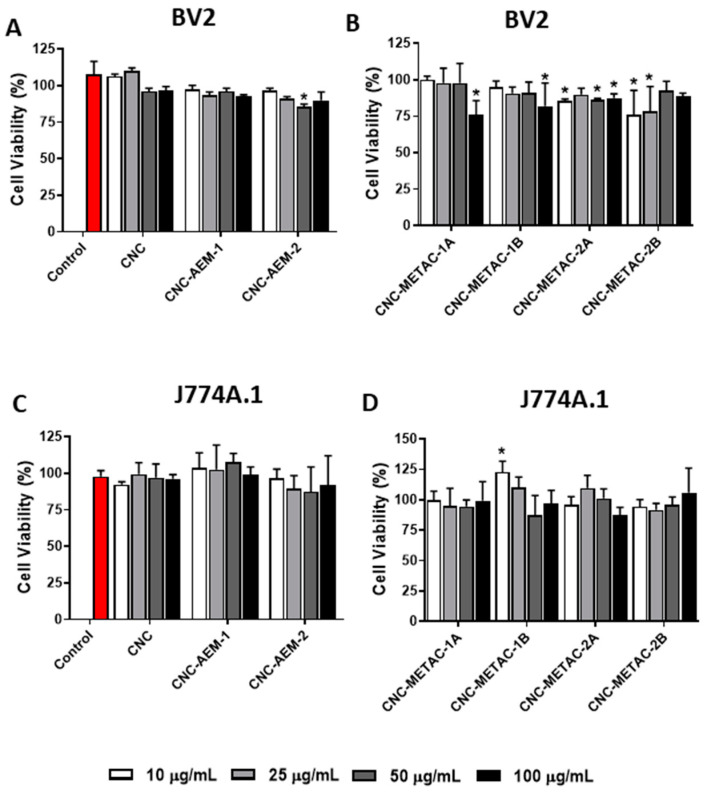
Cytotoxicity of unmodified and modified CNCs assessed by MTT assay. After 24 h of treatment, cell viability in BV2 and J774A.1 cell lines was determined spectrophotometrically. (**A**,**C**) pristine CNC and AEM modified and (**B**,**D**) METAC modified CNCs. Representative of 3 independent experiments, in triplicates. * *p* < 0.01 compared to control, non-treated cells.

Assessment of toxicity in cell-based assays is a rapid, simple, and affordable approach to address the initial safety of nanomaterials with potential for biomedical application. These cytotoxic assays can be considered as a crucial part of the characterization of nanomaterials [35]. To evaluate the cytotoxicity of the cationic CNCs, we chose to perform MTT and NR assays using the firmly attached J774A.1 and BV2 cell lines because (i) these assays are largely used for screening of toxic and harmless compounds, (ii) quick analysis and low manipulation of attached cells after treatment and (iii) satisfactory reproducibility. In addition to cell lines, we tested the toxicity in a more relevant cell system, such as human cells PBMCs since the biological application of these nanomaterials can involve systemic circulation throughout the body. To assess the CNCs impact on cell viability in PBMCs, which remain loosely attached to the plate when not primed, we chose LIVE/DEAD^®^ assay followed by flow cytometry analysis since it is an easy-to-use and sensitivity assay and PBMCs are not the most suitable cell type to access cytotoxicity by MTT or NR using a plate reader. Overall, our results indicated that CNCs have none or very low negative impact (<20%) on the cell viability assessed by all three cytotoxicity assays in all the cell types tested. However, results differed among CNCs and some cell types are more sensitive than others, depending on the assay used. Using MTT assay, which assesses cell viability by mainly mitochondria dehydrogenases, our data showed that in BV2 cells, the pristine CNC and CNC-AEM-1 did not decrease cell viability in any tested condition (Figure 3A). However, the METAC-modified CNCs did show a small decrease in cell viability, statistically significant, but not in a dose-response manner (Figure 3B). Using the same assay in J774A.1 cells, all the AEM-(Figure 3C) and METAC-modified CNCs (Figure 3D) did not impact negatively the cell viability in all tested concentrations. Interestingly, at the lowest concentration of CNC-METAC-1B (10 µg/mL), an increase in the conversion of tetrazolium to formazan was observed (Figure 3D). This effect does not indicate that there was an increase in cell viability above the control but rather an increase in the mitochondria dehydrogenase activity. This effect is not unknown since we have previously observed similar increases with other functionalized CNCs in other cell types, including MCF-7, a breast cancer cell line [23]. One reason for such an effect could be attributed to more tetrazolium dye entering into the cells, and therefore being more available to the dehydrogenases. A plausible explanation for potentially more dye entering the cells is that engineered nanomaterials, particularly high aspect ratio materials, can cause plasma membrane perturbation among other effects [36].

**Figure 4:**
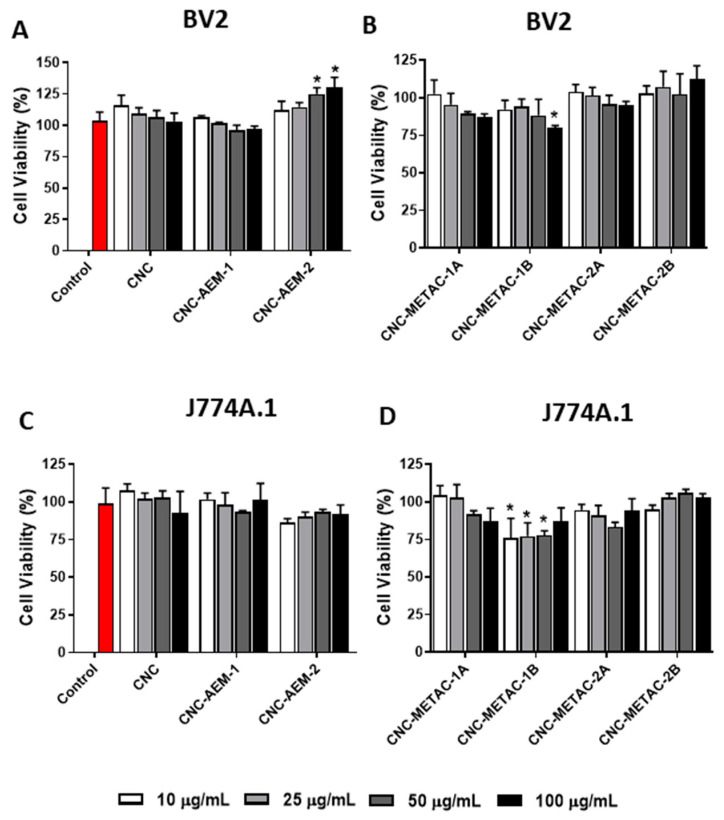
Cytotoxicity of unmodified and modified CNCs assessed by Neutral Red assay. After 24 h of treatment, cell viability in BV2 and J774A.1 cell lines was determined spectrophotometrically. (**A**,**C**) pristine CNC and AEM modified and (**B**,**D**) METAC modified CNCs. Representative of 3 independent experiments, in triplicates. * *p* < 0.01 compared to control, non-treated cells.

Data from NR assay indicated that in BV2 cells, CNC-AEM-2 appeared to cause an augmentation of NR dye uptake mainly in the highest concentrations (Figure 4A). A marginal negative effect on cell viability was observed in the cells treated with CNC-METAC-1B at the highest dose, 100 µg/mL (Figure 4B). Similar to what was observed with MTT assay, the J774A.1 cell line appeared to be less sensitive than BV2 cells. The AEM-modified CNC did not affect J774A.1 cells (Figure 4C) and only CNC-METAC-1B had a negative effect on the viability of these cells, although not a dose-response effect (Figure 4D). These differences could be attributed to potential physico-chemical changes on the nanomaterials since some nanomaterials might tend to form large agglomerates, sometimes larger than 100 nm size due to particle interaction with serum proteins [37], which was observed to have a significant effect on particle dispersion for certain materials. Furthermore, the cell lines used in this study were grown in a different medium, for instance, while J774A.1 cells were cultivated in RPMI, BV2 cells were grown in DMEM. Another aspect to consider is the differences in cellular responses was the biological differences among cell lines per se.

**Figure 5:**
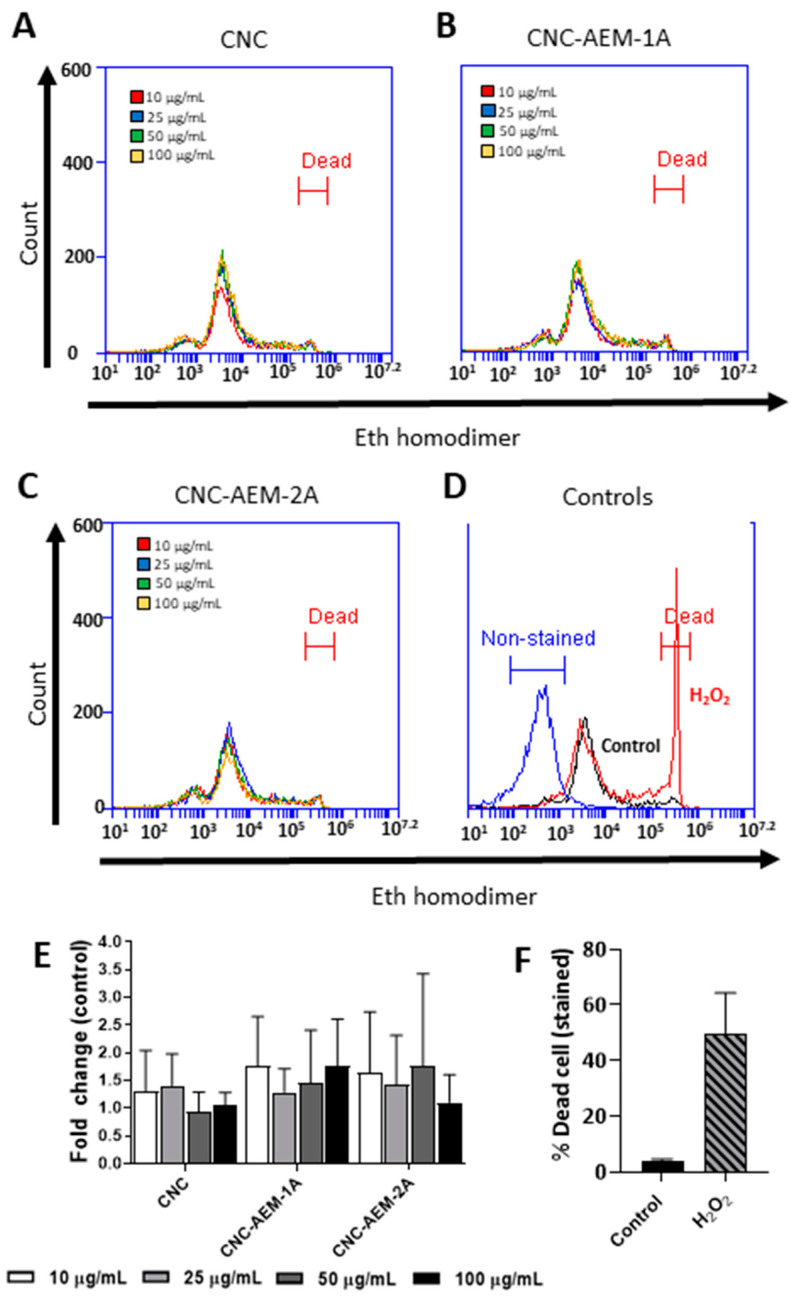
Flow cytometer plots from a representative experiment displaying the cytotoxic effects of unmodified (**A**) and AEM-modified CNCs (**B**,**C**) on PBMCs, as well as controls for gating (**D**). After 24 h of treatment, cell viability was determined by LIVE/DEAD^®^ assay. The graph (**E**) displayed the average of fold of change in the (%) of dead cells (ethidium bromide staining) corrected by (%) dead cells in the control (non-treated cells), from at least 4 experiments. H_2_O_2_ 500 µM, 4h was used as positive control for gating dead cells for the flow cytometer analysis (**D**,**F**).

**Figure 6:**
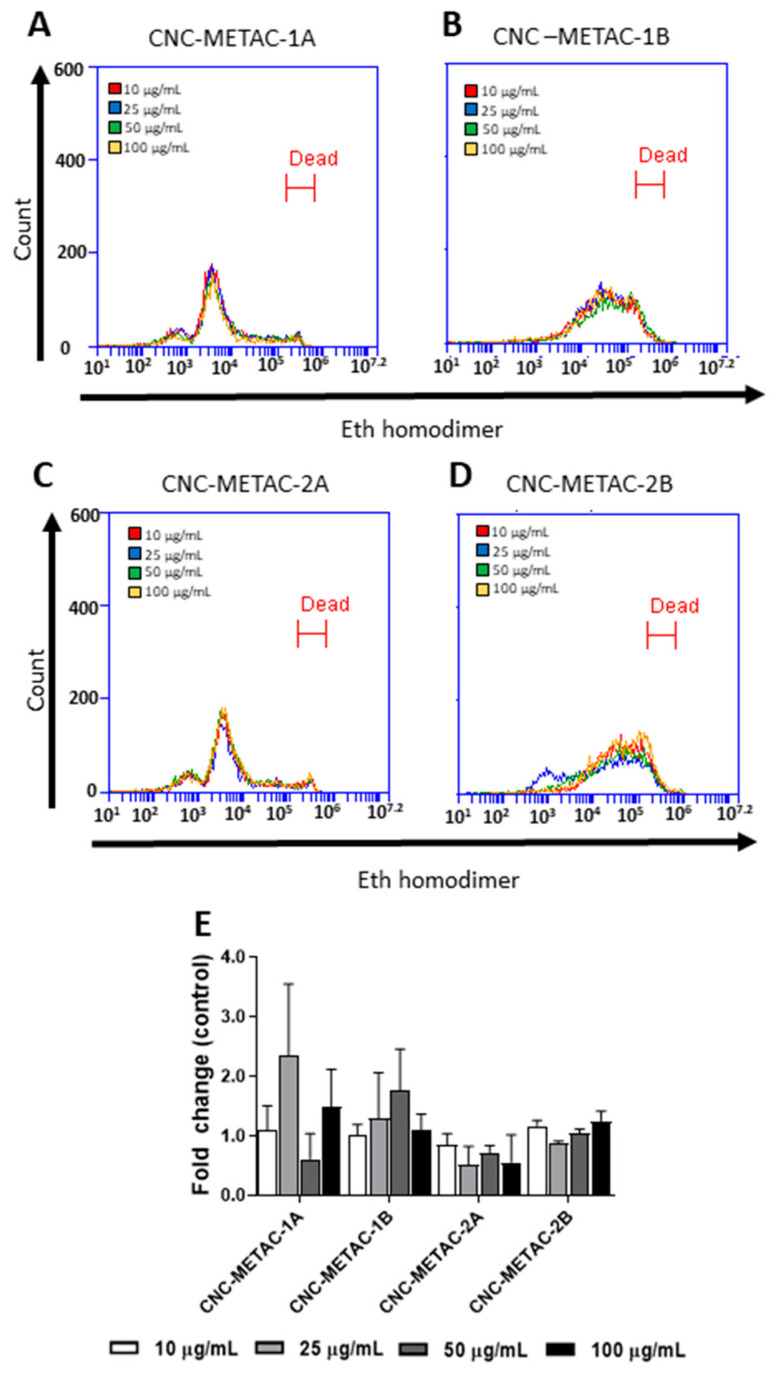
Flow cytometer plots from a representative experiment displaying the cytotoxic effects of METAC-modified CNCs (**A**–**D**) on PBMCs. After 24 h of treatment, cell viability was determined by LIVE/DEAD^®^ assay. The graph (**E**) displayed the average of fold of change in the (%) of dead cells (ethidium bromide staining) corrected by (%) dead cells in the control (non-treated cells), from at least 4 experiments.

We also evaluated the cytotoxicity of these modified CNCs using PBMCs and LIVE/DEAD^®^ assay. Corroborating with what was observed with cell lines, the overall cell viability in primary cells PBMCs was not affected significantly and in a dose-response manner. Despite the variation of the data, the percentage of ethidium bromide-labeled cells did not change significantly in the presence of different concentrations of AEM- or METAC-modified CNCs, in comparison to the non-treated cells (Figure 5 and Figure 6, respectively). As expected, H_2_O_2_ (positive control) increased the dead cell population as observed in the significant increases in the percentage of ethidium bromide labeling (Figure 5D,F). Although we observed that CNC-METAC-1B (Figure 6B) and CNC-METAC-2B (Figure 6D) displayed different flow cytometer spectrum from other CNCs, this difference appeared not to be related with an increase in cell death since it neither showed a characteristic peak in the H_2_O_2_ intensity range (Figure 5,D) nor a dose-response effect. We also did not observe differences in Calcein-AM staining between treatments and control. The SSC vs. FSC plot of non-stained cells was used for gating cells and excluding debris (Figure S6). A typical LIVE DEAD^®^ flow cytometer SSC vs. FSC plot of staining cells (Ethidium bromide and Calcein-AM) treated of 100 µg/mL is displayed in the supplemental materials (Figure S7).

## 4. Conclusions

In conclusion, a series of novel cationic CNCs were successfully synthesized via SI-SET-LRP at room temperature. The cationic CNCs displayed rod-like structures with different sizes and positive surface charges. The morphological features and structural integrity of the cationic CNCs were retained as evidenced by both TEM and AFM analyses. In general, the cationic CNCs showed low toxicity and the slight decreases in cell viability were cell-type dependent and did not indicate any correlation with physico-chemical characteristics. This initial analysis is crucial for further biological applications, suggesting that these cellulose-based nanomaterials would be good candidates to be investigated as immunomodulators and further developed as potential vaccine nanoadjuvants.

## Figures and Tables

**Table 1:** Different molar ratios of [Br]/[AGU] and [monomer]/[AGU] for the fabrication of cationic CNCs.

Sample	[Br]/[AGU]	[Monomer]/[AGU]
CNC-METAC-1A	5:3	50:3
CNC-METAC-1B	5:3	60:3
CNC-METAC-2A	5:12	50:3
CNC-METAC-2B	5:12	60:3
CNC-AEM-1A	5:3	50:3
CNC-AEM-2A	5:12	50:3

**Table 2:** Apparent particle size (DLS) and zeta potential of pristine and modified CNC samples.

Sample	Apparent Particle Size (nm)	Polydispersity Index (PdI)	Zeta Potential (mV)
Pristine CNCs	101.6 ± 0.72	0.23	−34.8 ± 2.16
CNC-BriB-1	96.3 ± 1.65	0.18	−28.9 ± 1.47
CNC-BriB-2	99.9 ± 1.80	0.22	−30.2 ± 1.69
CNC-METAC-1A	123.4 ± 1.32	0.20	+31.8 ± 2.89
CNC-METAC-1B	136.2 ± 1.50	0.22	+44.9 ± 3.93
CNC-METAC-2A	178.1 ± 2.53	0.24	+32.0 ± 0.94
CNC-METAC-2B	203.2 ± 2.66	0.31	+38.2 ± 0.94
CNC-AEM-1A	172.0 ± 9.08	0.38	+45.0 ± 1.44
CNC-AEM-2A	215.3 ± 2.86	0.31	+41.4 ± 3.15

**Table 3:** Elemental analysis of pristine and cationic CNCs.

Sample	% Carbon	% Hydrogen	% Nitrogen	% Sulfur
Pristine CNCs	40.92	6.07	0.00	0.30
CNC-METAC-1A	44.32	6.94	5.90	<0.20
CNC-METAC-1B	44.38	7.47	7.68	<0.20
CNC-METAC-2A	42.58	6.56	1.70	<0.20
CNC-METAC-2B	43.10	6.59	1.82	<0.20
CNC-AEM-1A	41.97	6.67	5.80	0.22
CNC-AEM-2A	40.45	6.35	4.83	<0.10

## Supplementary Materials

The following are available online at https://www.mdpi.com/2079-4991/10/8/1603/s1: Preparation of CNC-initiators and cationic CNCs, Figure S1: FTIR spectra of pristine CNCs, CNC-BriB-1 and CNC-BriB-2, Table S1: Amounts of reactants and reagents used for the preparation of cationic CNCs, Figure S2: FTIR spectra of cationic CNCs, Figure S3: Intensity-averaged size distribution profiles for pristine CNCs and CNC-METAC-1B in water, Figure S4: TEM images of cationic CNCs, Figure S5: AFM height and phase images of cationic CNCs, Figure S6: controls and gating for flow cytometry, Figure S7: Typical representative flow cytometry SSC vs. FSC plots.
