# Supplementary material for: Synthesis and Cytotoxicity Studies of Wood-Based Cationic Cellulose Nanocrystals as Potential Immunomodulators
Source: Nanomaterials (Basel). 2020 Aug 15;10(8):1603. doi: 10.3390/nano10081603 (PMC7466698; doi:10.3390/nano10081603)
Supplement: Supplementary file 1 [file nanomaterials-10-01603-s001.pdf]

Synthesis and cytotoxicity studies of wood-based cationic cellulose nanocrystals as potential immunomodulators

Yusha Imtiaz[a], Beza Tuga[a], Christopher W. Smith[a], Alexander Rabideau[a], Long Nguyen[a], Yali Liu[b], Sabahudin Hrapovic[b], Karina Ckless[a]*, Rajesh Sunasee[a]*

[a]Department of Chemistry, State University of New York at Plattsburgh, Plattsburgh, New York, USA, 12901.

[b]Aquatic and Crop Resource Development Research Centre, National Research Council Canada, Montreal, Quebec, Canada, H4P 2R2

*Authors for correspondence email: rajesh.sunasee@plattsburgh.edu & kckle001@plattsburgh.edu

Supporting information:

-Preparation of CNC-BriB-1 and CNC-BriB-2

-FTIR spectra and analysis of CNC-BriB-1 and CNC-BriB-2

-Synthesis of cationic CNCs

-FTIR spectra of cationic CNCs

- Dynamic light scattering of pristine CNCs and cationic CNCs

-TEM images of cationic CNCs

-AFM images of cationic CNCs

-Controls and gating for flow cytometry

-Typical representative flow cytometry SSC vs FSC plots.

Preparation of CNC-initiator (CNC-BriB-1 and CNC-BriB-2)

CNC-initiator was prepared according to a previously reported procedure [2]. Spray-dried CNCs were reacted with BriB initiator in a ratio of 5:3 and 5:12 with respect to anhydroglucose (AGU) units in CNCs to afford CNC-BriB-1and CNC-BriB-2 respectively. CNCs (1.00 g, 6.17 mmol of AGU, equivalent to 18.5 mmol OH groups) were dispersed in dry THF and stirred continuously in a 250 mL flask under N_2 gas at room temperature. TEA (2.3 mL, 16.5 mmol) was then added followed by DMAP (2.26 g, 18.5 mmol). BriB (1.25 mL, 10.0 mmol for CNC-BriB-1; 0.313 mL 2.50 mmol for CNC-BriB-2) was added dropwise and the resulting suspension was stirred for 24 h at room temperature. The mixture was centrifuged (10000 rpm at 10 °C for 10 min) twice with dry THF, twice with 1:1 THF:EtOH and once with acetone. The precipitate was allowed to dry at room temperature to yield a white powdery material.

Characterization of CNC-initiator by FTIR spectroscopy

FTIR spectra of pristine spray-dried sulfated CNCs and lyophilized esterified CNCs were conducted on a PerkinElmer FTIR spectrophotometer (Spectrum Two) at room temperature. KBr pellets were prepared by grinding in a mortar and compressing about 2% of the CNC samples in KBr (previously well-dried in an oven). Background measurement using a neat KBr pellet was first obtained to correct for light scattering losses in the pellet and any water absorbed by KBr. Spectra in the range of 4000-400 cm^{-1} were obtained with a resolution of 4 cm^{-1} by cumulating 32 scans.

<u>Analysis</u>: When compared to the spectrum of pristine CNCs, CNC-BriB-1 and CNC-BriB-2 indicated a new carbonyl ester peak at 1738 cm^{-1} and 1732 cm^{-1}, respectively (Figure S2). CNC-

BriB-1 has more initiator sites and as expected, the intensity of the carbonyl ester peak for CNC-BriB-1 was higher than CNC-BriB-2 with less initiator sites.

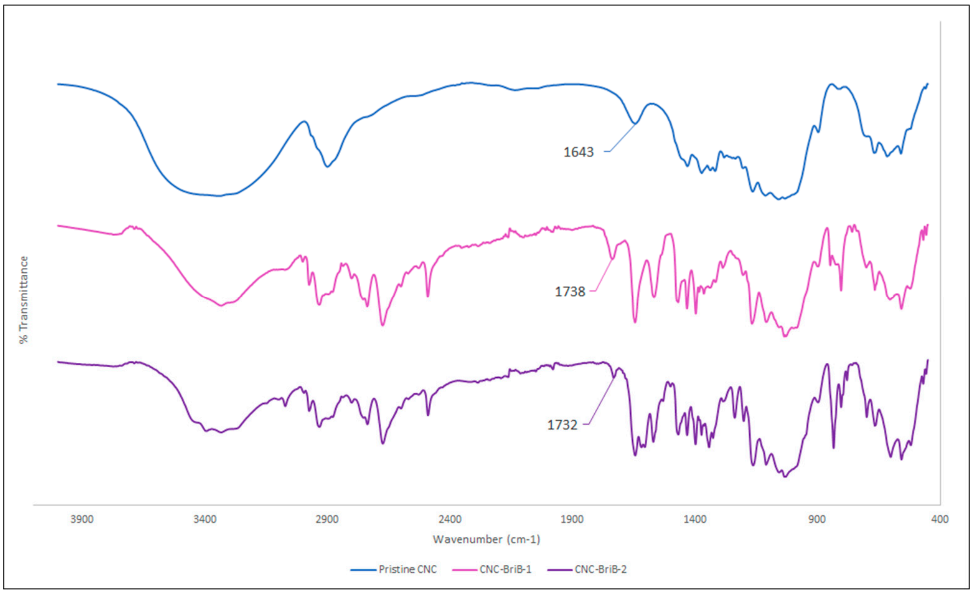

Figure S1: FTIR spectra of pristine CNCs, CNC-BriB-1 and CNC-BriB-2

General procedure for synthesis of cationic CNCs

CNC-BriB (300 mg) was dispersed in a MeOH:H_2O solvent mixture (100 mL, 1:1 v/v) in a 250 mL Schlenk flask. The reaction mixture was degassed under N_2 gas for 1 hr prior to the addition of the monomer METAC or AEM (50 and/or 60 mmol) and copper (I) bromide (0.5 mmol). The suspension was degassed again before addition of bpy or PMDETA (1 mmol) and the reaction mixture was vigorously stirred at room temperature for 24 h. The resulting crude cationic CNCs was then centrifuged (3 x 12000 rpm at 10 °C for 30 min) with a 1:3 ratio of H_2O:MeOH. The residual solid was resuspended in water and extensively dialyzed (MWCO 3500) against deionized water for 1 week with daily constant change of water. The suspension was then freeze-dried to afford purified CNCs as white flaky solid. Table S1 below showed the different amounts of reactants, monomers and reagents used for the synthesis of the cationic CNCs.

Table S1: Amounts of reactants and reagents used for the preparation of cationic CNCs

**Cationic CNCs**	**CNC-BriB-1**	**CNC-BriB-2**	**METAC**	**AEM**	**CuBr**	**bpy or PMDETA**
CNC-METAC-1A	350 mg	-	50 mmol	-	72 mg	bpy: 156 mg
CNC-METAC-1B	350 mg	-	60 mmol	-	72 mg	bpy: 156 mg
CNC-METAC-2A	-	350 mg	50 mmol	-	72 mg	bpy: 156 mg
CNC-METAC-2B	-	350 mg	60 mmol	-	72 mg	bpy: 156 mg
CNC-AEM-1A	350 mg	-	-	50 mmol	72 mg	PMDETA: 173 mg
CNC-AEM-2A	-	350 mg	-	50 mmol	72 mg	PMDETA: 173 mg

FTIR spectroscopy of cationic CNCs

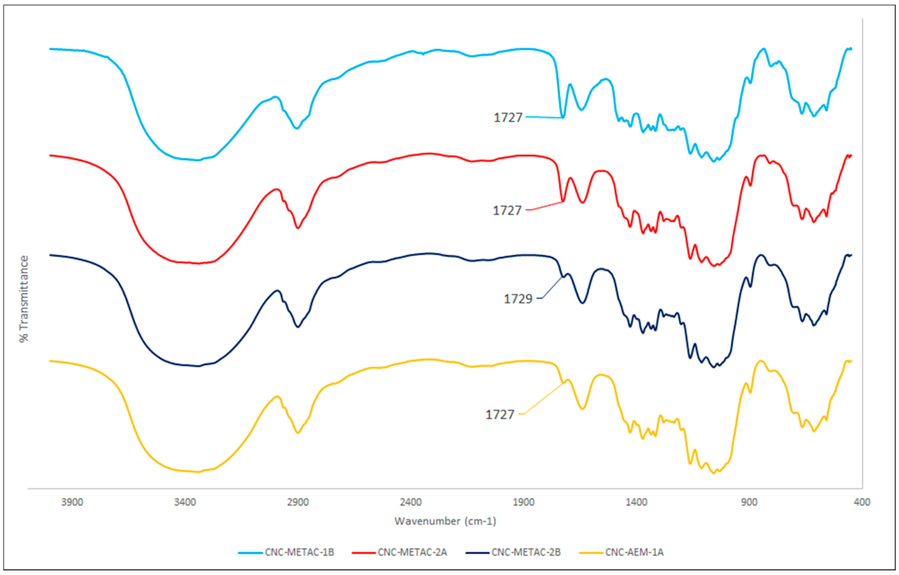

Figure S2: FTIR spectra of cationic CNCs: From top to bottom: CNC-METAC-1B, CNC-METAC-2A, CNC-METAC-2B and CNC-AEM-1A

Dynamic light scattering of pristine CNCs and CNC-METAC-1B

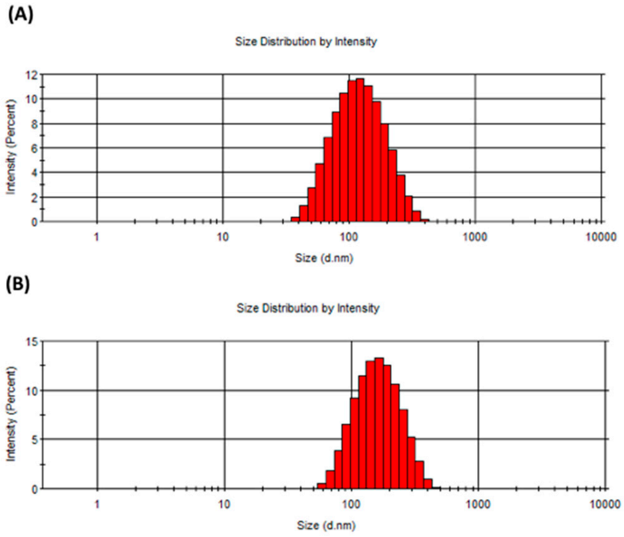

Figure S3: Intensity distribution profiles for (A) pristine CNCs and (B) CNC-METAC-1B in water (0.05 wt%)

TEM images of cationic CNCs

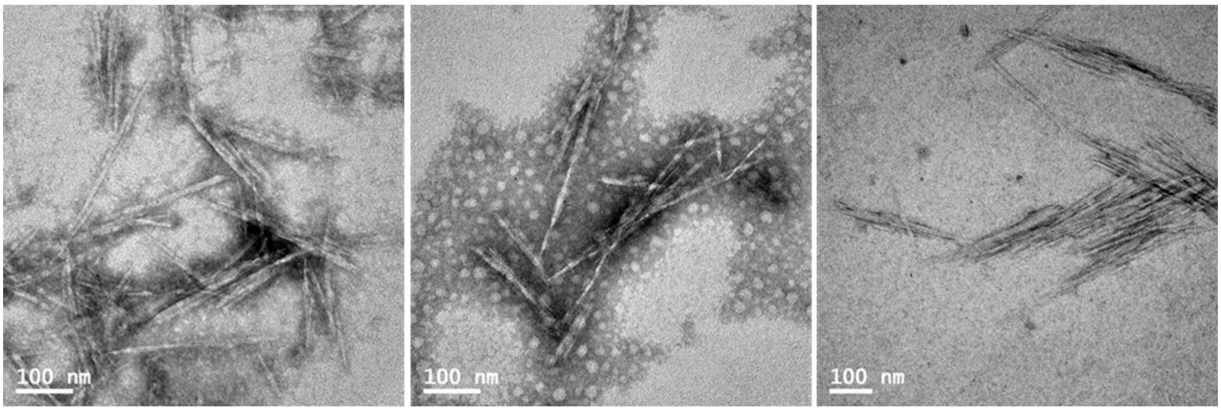

Figure S4: TEM images of cationic CNCs (from left to right): CNC-METAC-2A; CNC-METAC-2B; CNC-AEM-2A

AFM images of cationic CNCs

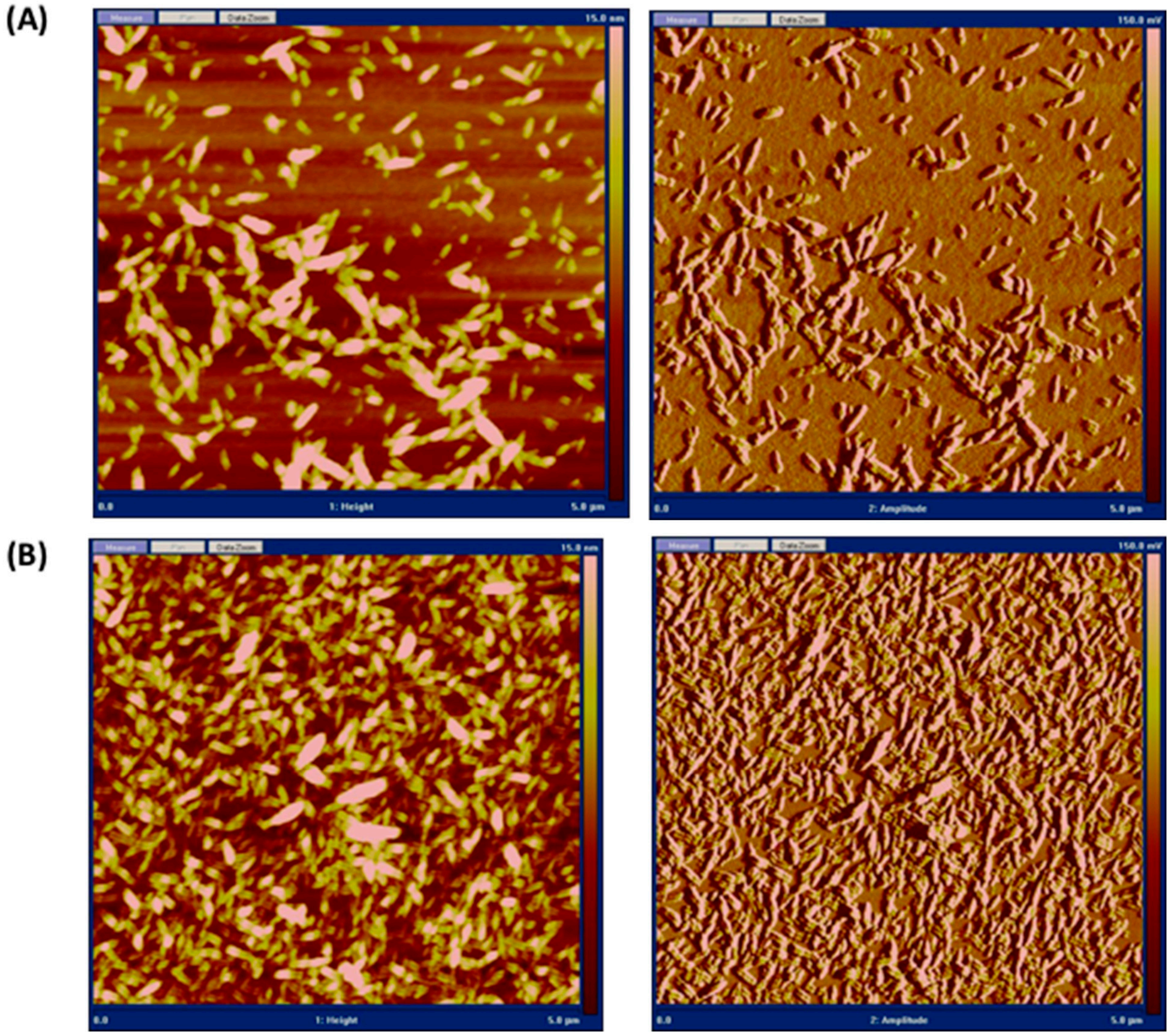

Figure S5: Height images (left) and phase images (right) of cationic CNCs: (A) CNC-METAC-1A; (B) CNC-METAC-1B

(A) **Gating – non-stained**

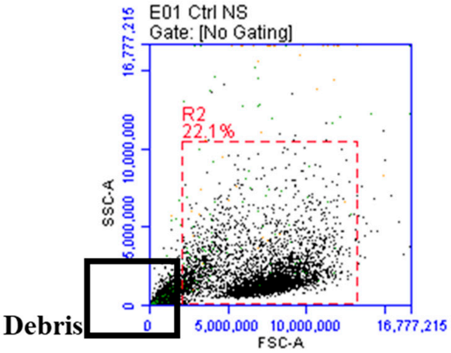

(B) **Ethidium bromide and Calcein-AM staining controls – non treated cells**

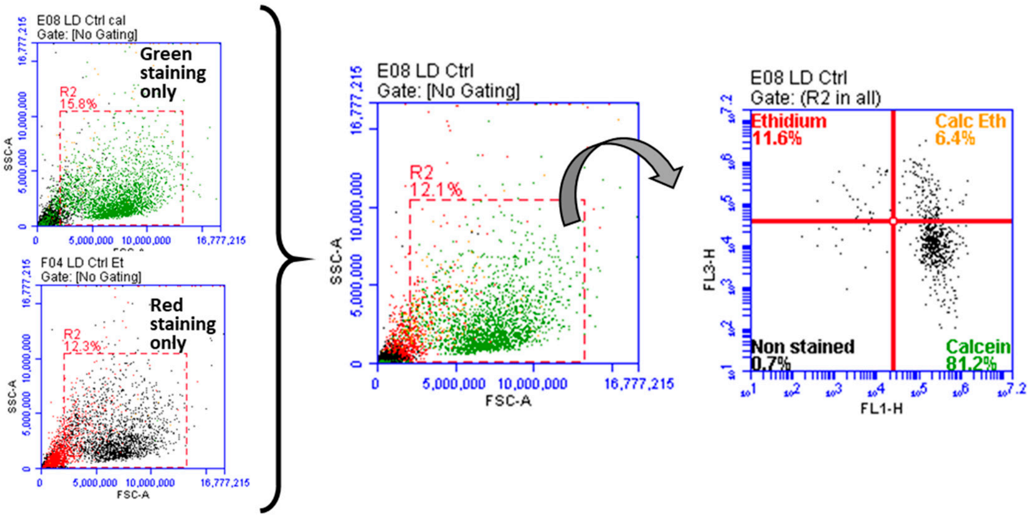

Figure S6: Typical representative plots for gating and staining control for flow cytometry analysis. (A) SSC vs FSC density plot of non-stained cells demonstrating the gate (R2) that has been applied to identify a specific population, in this case lymphocytes, and remove debris (black square). (B) Calcein-AM, green staining only or ethidium bromide, red staining only in control (non-treated cells). This gating (R2) was further applied to identify red and green staining populations in cells treated with CNCs.

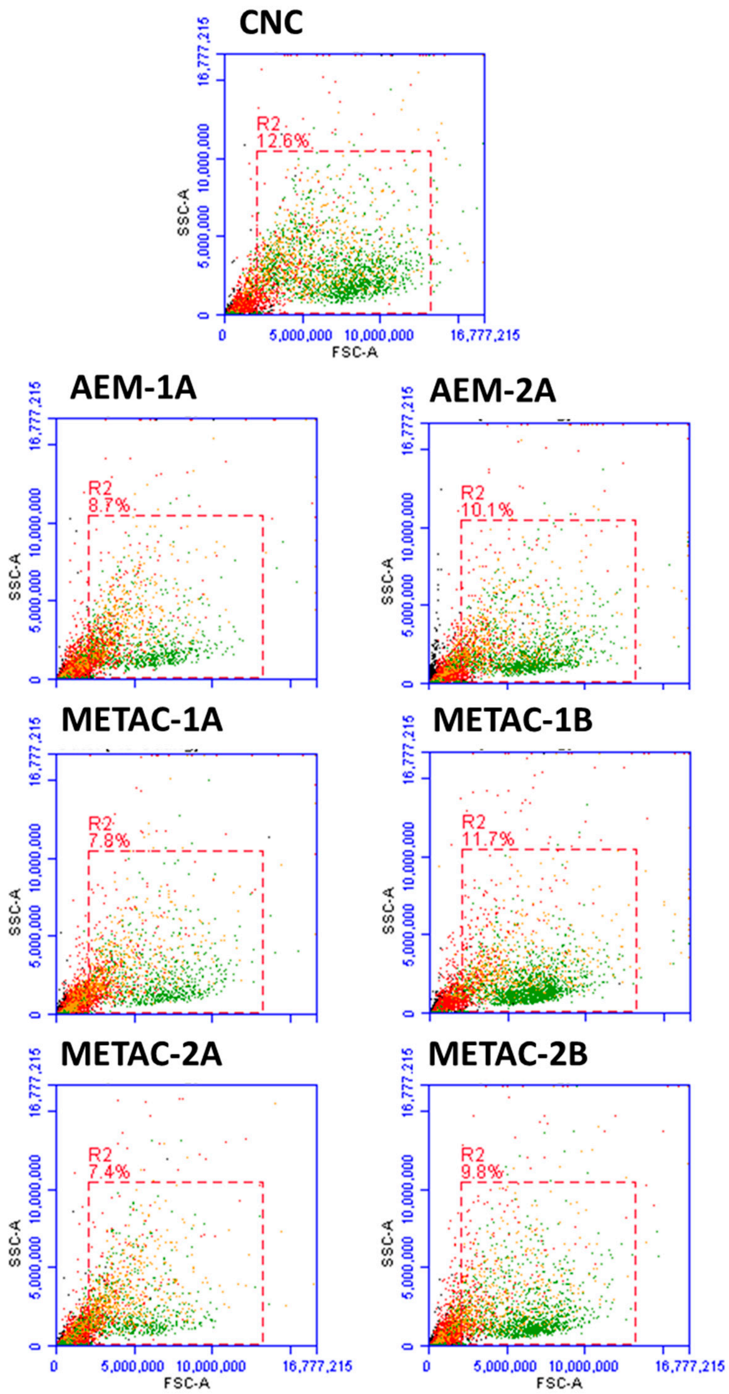

Figure S7: Typical representative SSC vs FSC plots indicating the cellular gating as well as the red and green staining populations in cells treated with CNCs 100 µg/mL. The gating (R2) for cells of interest was determined in non-stained cells (Figure S6A).

Reference

1. Hemraz, U.D.; Campbell, K.A.; Burdick, J.S.; Ckless, K.; Boluk, Y.; Sunasee, R. Cationic Poly(2-aminoethylmethacrylate) and Poly(N-(2-aminoethylmethacrylamide) Modified Cellulose Nanocrystals: Synthesis, Characterization, and Cytotoxicity. *Biomacromolecules*. **2015**, *16*, 319-325.
